# Effects of Graphene Oxide on the Growth and Photosynthesis of the Emergent Plant *Iris pseudacorus*

**DOI:** 10.3390/plants12091738

**Published:** 2023-04-23

**Authors:** Zixin Zhou, Jiaxin Li, Cui Li, Qiang Guo, Xincun Hou, Chunqiao Zhao, Yu Wang, Chuansheng Chen, Qinghai Wang

**Affiliations:** 1 College of Environmental Science and Engineering, Central South University of Forestry and Technology, Changsha 410004, China; 2 Institute of Grassland, Flowers and Ecology, Beijing Academy of Agriculture and Forestry Sciences, Beijing 100097, China; 3 State Key Laboratory of Multiphase Complex Systems, Institute of Process Engineering, Chinese Academy of Sciences, Beijing 100190, China

**Keywords:** aquatic plant, graphene oxide, photosynthesis, plant nutrients

## Abstract

The extensive applications of graphene oxide (GO) inevitably lead to entry into the natural aquatic environment. However, information on its toxicity to emergent plants is still lacking. In this study, an emergent plant, *Iris pseudacorus,* was exposed to GO (1, 20, 80, and 140 mg·L^−1^) under hydroponic conditions for 15 weeks. Changes in plant growth were assessed by analyzing plant biomass and photosynthetic pigment contents; the photosynthesis response was verified by measuring chlorophyll *a* fluorescence; and the nutrient levels of the plant were evaluated. Results showed that GO at 20–140 mg·L^−1^ significantly increased plant dry weight by 37–84% and photosynthetic pigment contents by 26–178%, and 80 mg·L^−1^ was the optimal concentration. PSII activity, adjustment capacities of electron transport in PSII, the grouping or energetic connectivity between PSII units, light energy conversion efficiency, photosynthesis performance indexes (by 11–51%), and contents of several nutrient elements (N, Fe, and Cu) were increased by 49–69%, 34–84%, and 11–38%, respectively. These findings indicate that GO can enhance plant growth by promoting plant photosynthesis performance and improving plant nutrient levels, and has great application potential in promoting the growth and development of this emergent plant as a phytoremediation agent.

## 1. Introduction

Graphene oxide (GO) is one of the most popular and representative two-dimensional carbon-based nanomaterials, and has been widely used in various fields [1,2]. Due to its large-scale production and widespread application, its inevitable release to the natural aquatic environment and subsequent potential risk to aquatic ecosystem have aroused increasing attention worldwide [3,4,5]. The ecotoxicological impacts of GO have been estimated on various model and non-model aquatic organisms. GO was able to accumulate and induce significant oxidative stress in *Chironomus riparius*, a sentinel species in the freshwater ecosystem [6]. It also had toxic effects on the fish *Anabas testudineus*, evidenced by augmented accumulation of lipid peroxides and activities of antioxidant enzymes, as well as alleviated total red blood corpuscle counts and protein levels [7]. In addition, GO caused physical injury and endocrine-disrupting effects on fish because of its sharp edges and high adsorptive property [8]. GO also posed similar indirect toxicity to the algae *Microcystis aeruginosa* through physical mechanisms associated with light shading and cell aggregation [9].

Owing to its higher water dispersity and transformation, as well as abundant functional groups, GO at 50 mg·L^−1^ inhibited the growth of fresh algae (*Chlorella pyrenoidosa*) by the shading effect, oxidative stress-induced membrane damage, and nutrient depletion after exposure for 96 h [10]. However, using the aeroterrestrial microalga *Trebouxia gelatinosa* as an assessed species, both long-term (4 weeks) and short-term (10 or 30 min) exposures to GO at 50 mg·L^−1^ had no negative effect on the viability of the algae, as its thick cell wall effectively impeded the internalization of GO in the cell, thus preventing interferences with the cytoplasm [11]. A recent study further confirmed that the toxicity of GO varied in different algal species, and oxidative stress and membrane damage were the major toxic mechanisms. Physiological characteristics, cell migration, and cell wall composition of the algae also had significant effects on GO toxicity [12]. In agreement with the above observations, a study on bacteria showed that oxidative stress mediated the cellular toxicity of GO; thus, GO still led to indirect cytotoxicity via mechanical damage to cells or interfering with the cell metabolism, even though it did not enter the cells. Therefore, controlling the GO reduction and maintaining its solubility are possible ways of minimizing the toxicity of GO [13]. Similarly, oxidative damage, accompanied by a reduction in shoot biomass, was observed in rice (*Oryza sativa* L.) exposed to GO at 100 and 250 mg·L^−1^, and the surface oxygen content of GO was mainly responsible for these biological impacts [14]. Another study also revealed that the toxic mechanism of GO action was dependent on its surface oxidation [15]. These findings suggest that GO had similar toxic mechanisms in different species of aquatic organism. For more detailed and extensive information on the ecotoxicity of GO, a series of toxicity assays on nine aquatic organisms indicated that GO showed low acute toxicity for the aquatic bioindicator organisms, and its predicted no-effect concentration values were estimated as 20 to 100 μg·L^−1^. The planktonic microcrustacean *Daphnia magna* was particularly susceptible to exposure to GO, while the aquatic plant *Lemna minor* had a stronger tolerance against GO effects [16].

The different extents of GO toxicity in different aquatic organisms highlights a growing need for the investigations to be extended to a broader spectrum of primary producers in aquatic ecosystems. Moreover, considering the persistent existence of GO in aquatic environments, long-term investigations should be employed to more precisely predict the potential toxicity of GO in aquatic organisms [17]. Despite numerous studies which have been conducted to assess GO toxicity in aquatic environments, most investigations have focused on freshwater fish [7,8] and algae [9,10,11,12] in short-term exposure experiments; higher aquatic plants, especially emergent rooted plants in long-term exposure assessment, have seldom been taken into consideration. The emergent rooted aquatic plants are the main primary producers in aquatic ecosystems, and play an important role in the improvement of water quality and clarity by damping wave activity, stabilizing sediments, and, therefore, reducing shoreline erosion [18]. *Iris pseudacorus*, a typical emergent plant with high ornamental value, can withstand a broad range of environmental conditions [19]. Owing to its broad ecological tolerance and higher efficiency in pollutant removal, the plant is frequently used for urban landscaping [20] and phytoremediation of contaminants in water, such as organic compounds [21], heavy metals [20], and inorganic nutrients [5]. Additionally, planted constructed wetlands with *I. pseudacorus* enhanced phosphorus removal under GO exposure [5], and GO transformation showed a reduction in the constructed wetland [17].

For the purpose of bridging the knowledge gap on the toxicity of GO to emergent plants after long-term exposure, *I. pseudacorus* was selected as a model plant in the present study, and was exposed to GO at different levels under hydroponic conditions for fifteen weeks. The changes in total biomass and photosynthetic pigment contents of *I. pseudacorus* upon exposure to GO were investigated to clarify the influence of GO on plant growth. Further, plant photosynthesis and nutrient levels were determined, which may help to clarify the possible influencing mechanism of GO on plant growth. These observations provide the basic information needed to more precisely predict the potential toxicity of GO to aquatic organisms, and to explore the potential of the emergent plant to be used as a phytoremediation agent combined with GO for pollutant removal in water.

## 2. Results and Discussion

### 2.1. Biomass and Photosynthetic Pigment Contents of I. pseudacorus

**Figure 1:**
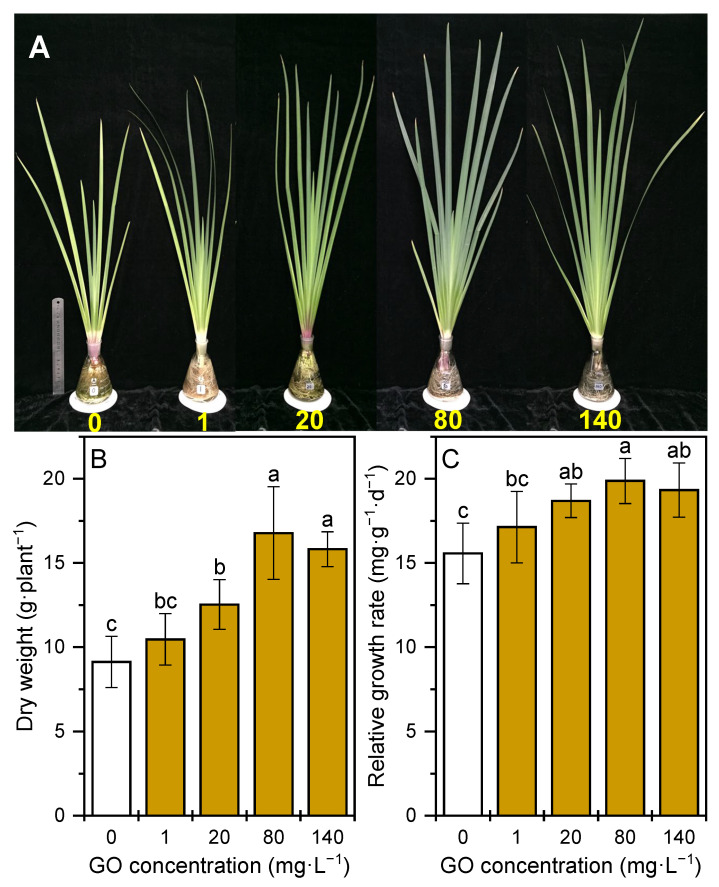
(**A**) Optical photograph, (**B**) dry weight, and (**C**) relative growth rate of *I. pseudacorus* under either the control or GO treatment for 15 weeks. All data are expressed as means ± SD (n = 4). Different letters indicate significant differences between different GO concentrations according to Duncan’s test (*p* < 0.05).

The photosynthetic pigments in plants are the main substances responsible for light-harvesting and energy transfer in photosynthesis. Variation in their contents is an important indicator of plants undergoing environmental stress. In this study, chlorophyll content, carotenoid content, and chlorophyll *a*/*b* of *I. pseudacorus* treated with GO increased by 26–178%, and the increase amplitude showed a clear rising trend with the increase in GO concentrations (Table 1). Chlorophyll *a* is considered as a light-harvesting pigment and as the reaction center of leaf photosynthesis; chlorophyll *b* can act as an auxiliary light-harvesting pigment and help chlorophyll *a* in leaf photosynthesis [22]. Carotenoids not only act as accessory light-harvesting pigments, but also perform an essential photoprotective role by quenching excess light energy and scavenging ROS formed within the chloroplast [23]. Thus, it can be seen that the promotion of photosynthetic pigments in plant driven by GO provided a basis for better capturing light energy and more effectively protecting the plant from the predicted stress. Inconsistently, it has been reported that GO caused reductions in the chlorophyll biosynthesis of *I. pseudacorus* in constructed wetlands; however, the strong increase in antioxidants, together with the slightly affected performance of the constructed wetland in pollutant removal, indicated that the plant was a GO-tolerant species [24]. Plant biomass can directly provide more useful information on plant growth. The plant treated with GO exhibited better development (Figure 1A). GO at 20, 80, and 140 mg·L^−1^ significantly increased dry weight by 37–84% (Figure 1B) and relative growth rate by 10–28% (Figure 1C) in *I. pseudacorus*. Similar results were obtained by a previous study, which found that GO at 25 mg·L^−1^ had stimulating effects on the growth of a free-floating aquatic plant, *L. minor* [15].

### 2.2. Chlorophyll Fluorescence Rise Kinetics OJIP Curves

**Figure 2:**
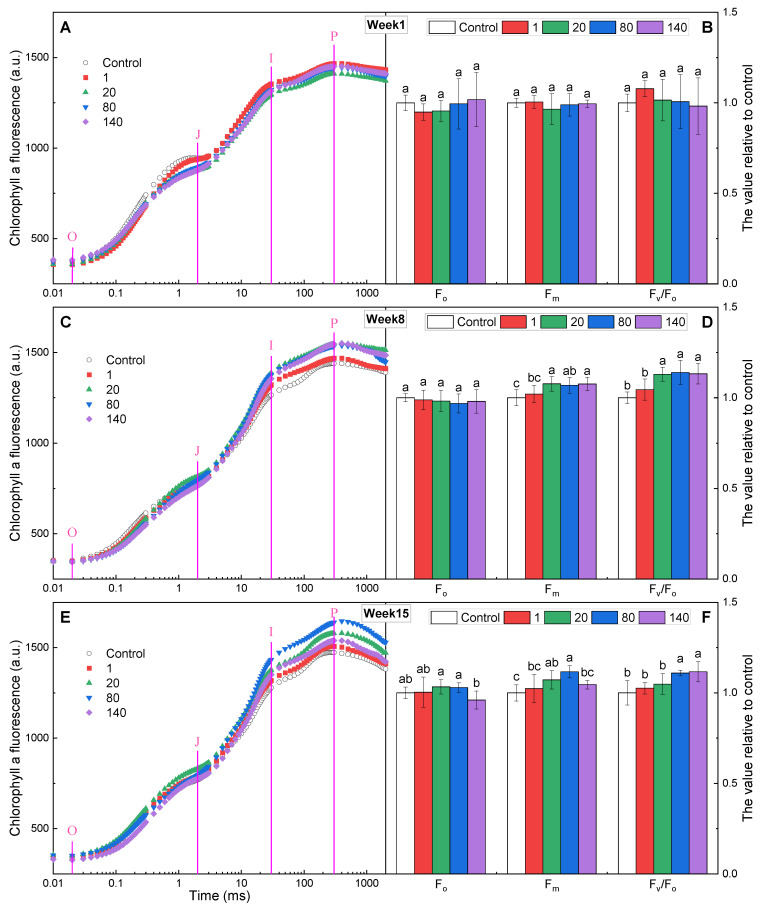
Raw chlorophyll a fluorescence OJIP transient curves, and values of F_o_, F_m_, and F_v_/F_o_ in *I. pseudacorus* treated with GO for (**A**,**B**) 1 week, (**C**,**D**) 8 weeks, and (**E**,**F**) 15 weeks. All data are expressed as means ± SD (n = 4). Each parameter followed by a different letter indicates a significant difference between different GO concentrations according to Duncan’s test (*p* < 0.05).

The kinetics of the rise in fluorescence brought on by chlorophyll *a* are highly sensitive to various environmental changes, and can provide both qualitative and quantitative information on the physiological state of photosynthetic apparatuses, especially photosystems (PS) II [25]. In the first week of culture, the J step of the rapid chlorophyll fluorescence kinetic curve (OJIP curve) at each GO concentration (except for 1 mg·L^−1^) was lower than that of the control (Figure 2A), but the initial fluorescence (F_o_), maximum fluorescence (F_m_), and PSII potential activity (F_v_/F_o_) did not significantly differ from the control (Figure 2B). In the 8th and 15th weeks of culture, the I and P steps increased significantly compared to the control (Figure 2C,E). F_o_ was not significantly different from the control, while F_m_ increased at 80 and 140 mg·L^−1^, with F_v_/F_o_ significantly increasing by 11–14% (Figure 2D,F). The J step reflected light-driven accumulation of the reduced primary quinone electron acceptor (Q_A_^−^) with the secondary quinone acceptor (Q_B_) being oxidized; the I and P steps reflected light-driven accumulation of Q_B_^−^ and Q_B_^2−^, respectively [26]. F_m_ reflected the state of electron transfer at the PSII donor side. The decrease in the J step with high F_m_, as well as the increases in the I and P steps, indicated that GO was able to facilitate electron transfer from Q_A_^−^ to Q_B_. F_v_/F_o_ represented the activity of the water-splitting complex on the donor side of PSII, and was shown to be a sensitive component in the photosynthetic electron transport chain [27]. The enlarged F_v_/F_o_ was mainly due to the increase in F_v_ in this study, suggesting an improvement in the efficiency of excitation energy capture by open reaction centers (RCs). Thus, the activity of the PSII water-splitting complex was enhanced.

**Figure 3:**
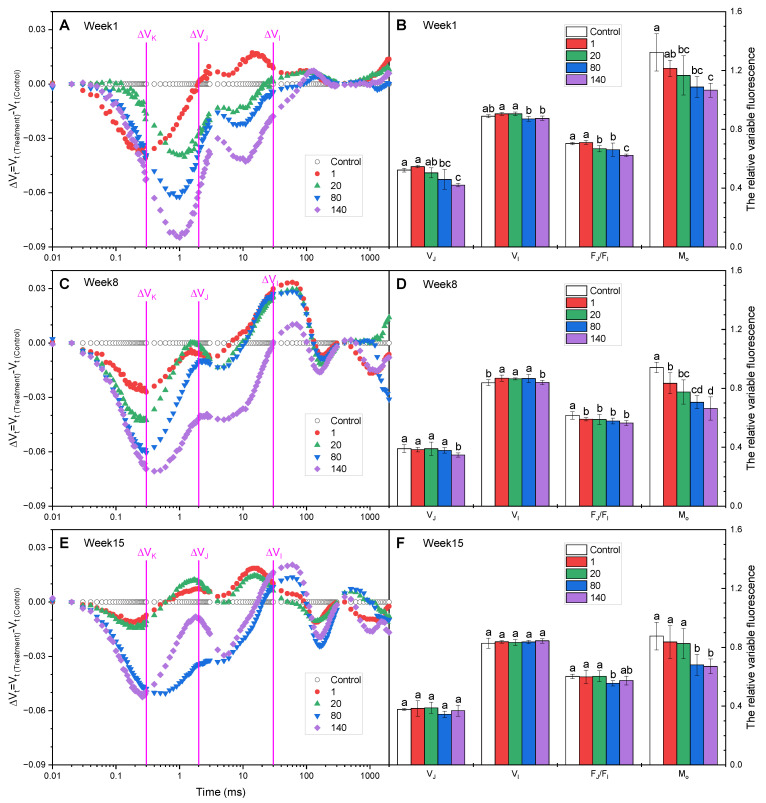
The relative variable fluorescence induction kinetic curves on a logarithmic time scale, and the values of V_J_, V_I_, F_J_/F_I_, and M_O_ in *I. pseudacorus* treated with GO for (**A**,**B**) 1 week, (**C**,**D**) 8 weeks, and (**E**,**F**) 15 weeks. All data are expressed as means ± SD (n = 4). Each parameter followed by a different letter indicates a significant difference between different GO concentrations according to Duncan’s test (*p* < 0.05).

ΔV_t_, at the logarithmic time scale, can show invisible features of OJIP curves [25]. The negative ΔV_K_ indicated that the fluorescence intensity at the K step was significantly lower in GO-treated plants compared with the controls (Figure 3A,C,E), which may indicate an improvement in the activities of the oxygen-evolving complex (OEC) on the electron donor side of PSII [28]. The relative variable fluorescence at the J step (V_J_) in plants treated with 140 mg·L^−1^ GO also decreased significantly in the 1st and 8th weeks, while no significant change was observed in 15th week. V_J_ in plants treated with 80 mg·L^−1^ GO decreased significantly only in the first week; F_J_/F_I_ in plants treated with 80 and 140 mg·L^−1^ was significantly lower than that of the control, indicating that GO decreased the J-step level. V_J_ was a measure of the fraction of the primary quinone electron acceptor of PSII that was in its reduced state [Q_A_^−^/Q_A(total)_], reflecting the accumulation of Q_A_^−^ [29,30]. The decrease in the value of V_J_ with increasing GO concentrations within eight weeks indicated the enhancement of Q_A_^−^ reoxidation capacity and the promotion of electron transfer at the donor side of PSII driven by GO. The initial slope at the beginning of the relative variable fluorescence transients (M_o_) is a measure of the relative rate of photochemistry, expressing the net rate of the RCs’ closure, which is associated with the rate of excitation capture and the photochemical efficiency of the first charge separation [29,31]. In the present study, the values of M_o_ in plants treated with GO were lower than those of the controls, and the difference was significant in plants treated with GO at 80 and 140 mg·L^−1^ (Figure 3B,D,F), suggesting the acceleration of electron flow [32]. This result was also supported by the data on V_J_ mentioned above.

### 2.3. The Initial Slope of the Standardized Fluorescence Transient F_t_/F_O_

**Figure 4:**
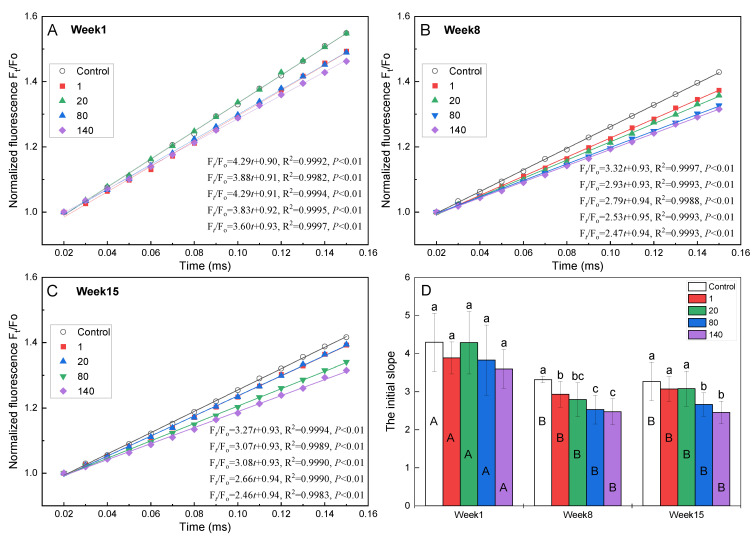
The OJIP transients, normalized by F_O_ as F_t_/F_O_, on a linear time scale from 0.02 to 0.15 ms in *I. pseudacorus* treated with GO for (**A**) 1 week, (**B**) 8 weeks, and (**C**) 15 weeks. (**D**) The initial slope of the standardized OJIP transient. All data are expressed as means ± SD (n = 4). Different lowercase letters indicate significant differences between different GO concentrations according to Duncan’s test, and different uppercase letters indicate significant differences between different weeks according to Duncan’s test (*p* < 0.05).

The initial slope of the standardized fluorescence transient (the fluorescence values are expressed as F_t_/F_o_) can provide detailed information on the rate of the electron transfer from P_680_ to Q_A_ [33]. For GO-treated plants, the initial slope was lower than that of the control, and exhibited an obvious downward trend with increasing GO concentrations (Figure 4); the difference was significant in the 8th and 15th weeks (Figure 4D). Moreover, for the same GO concentration, the initial slope in the 1st week was significantly higher than those in the 8th and 15th weeks (Figure 4D). These results suggested that GO limited the rate of electron transfer from P_680_ to Q_A_, and, therefore, reduced the accumulation of Q_A_^−^. The accumulation of excess electrons in the electron transport chain gave rise to the formation of reactive oxygen species (ROS), which could damage PSII [34]. The facilitation of electron transfer from Q_A_^−^ to Q_B_, mentioned above, can also reduce Q_A_^−^ accumulation. These changes demonstrated the improved ability of the plant to regulate and match electron transport capacity according to what is required for optimal photosynthesis performance.

### 2.4. K-Band and L-Band

**Figure 5:**
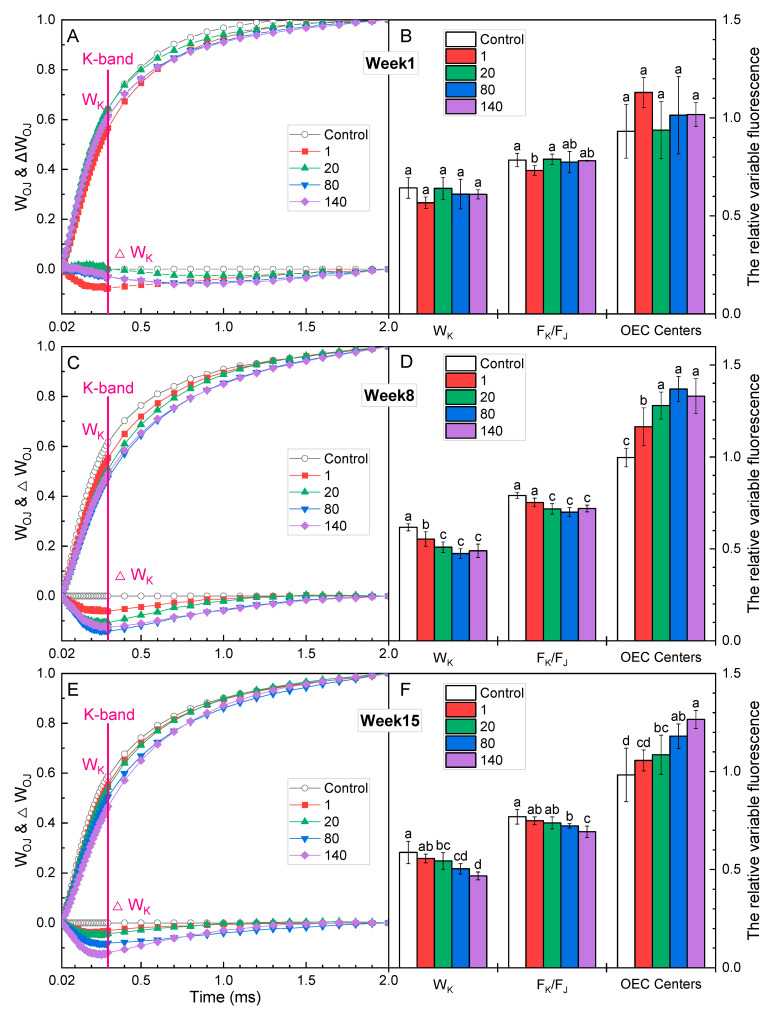
The fluorescence rise kinetics, normalized by F_o_ and F_J_ as W_OJ_, on a linear time scale from 0.02 to 2 ms, and the values of W_K_, F_K_/F_J_, and OEC centers in *I. pseudacorus* treated with GO for (**A**,**B**) 1 week, (**C**,**D**) 8 weeks, and (**E**,**F**) 15 weeks. All data are expressed as means ± SD (n = 4). Each parameter followed by different letters showed significant differences between different GO concentrations according to Duncan’s test (*p* < 0.05).

In the first week, there were no significant differences in the K-band between the GO treatments and the control (Figure 5A). The W_K_, F_K_/F_J_, and OEC centers also did not show significant differences (Figure 5B). In the 8th and 15th weeks, the K-band, W_K_, and F_K_/F_J_ of each GO concentration treatment were significantly lower than those of the control (Figure 5C,E), and the OEC center was significantly higher than that of the control (Figure 5D,F). OEC centers represent the active fraction of OEC [25]. The lower W_K_ and higher OEC centers in plants which received GO treatments indicated that electron transfer capacity on the donor side of PSII was enhanced, and this enhancement may be related to the increase in OEC content [35].

**Figure 6:**
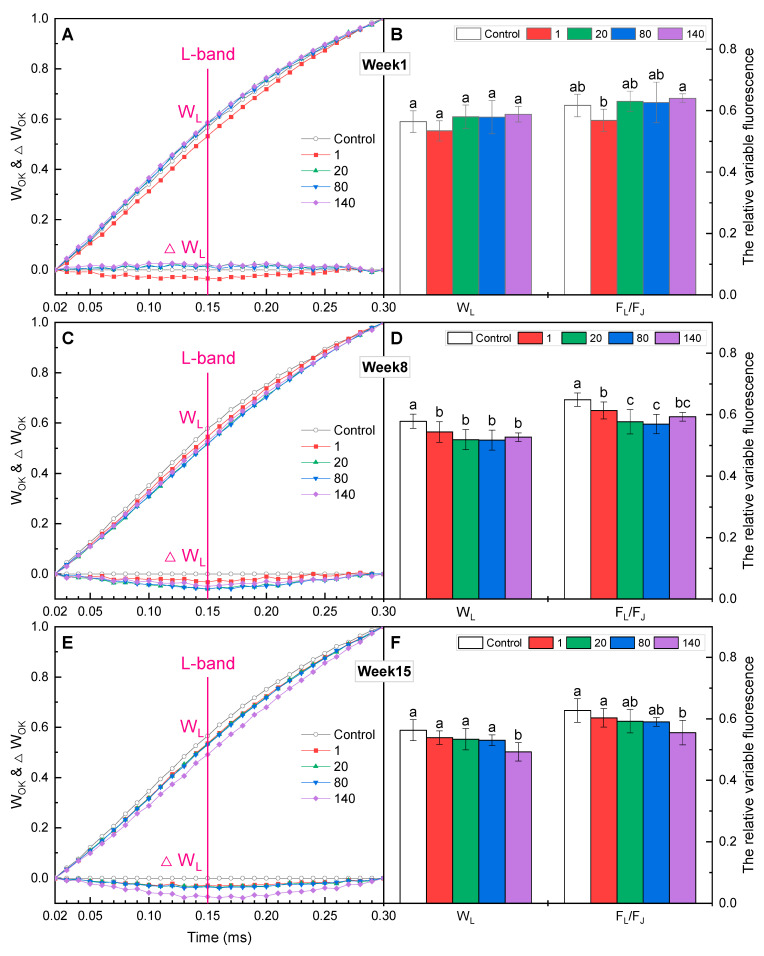
The fluorescence rise kinetics, normalized by F_O_ and F_K_ as W_OK_, on a linear time scale from 0.02 to 0.3 ms, and the values of W_L_ and F_L_/F_J_ in *I. pseudacorus* treated with GO for (**A**,**B**) 1 week, (**C**,**D**) 8 weeks, and (**E**,**F**) 15 weeks. All data are expressed as means ± SD (n = 4). Each parameter followed by different letters showed significant differences between different GO concentrations according to Duncan’s test (*p* < 0.05).

For the L-band, only GO at 1 mg·L^−1^ decreased in the first week (Figure 6A), while W_L_ and F_L_/F_J_ showed no significant difference from the control (Figure 6B). In the 8th and 15th weeks, an obvious negative L-band was detected in GO-treated plants (Figure 6C,E), and significantly lower W_L_ and F_L_/F_J_ were observed, especially for those who received GO at higher concentrations (Figure 6 D,F). The L-band is an indicator of the grouping of the PSII units or energetic connectivity between the antenna and PSII [25]. The significant decreases in the values of W_L_ and F_L_/F_J_ indicated a rise in energetic connectivity among PSII units.

### 2.5. Chlorophyll a Fluorescence Parameters

**Figure 7:**
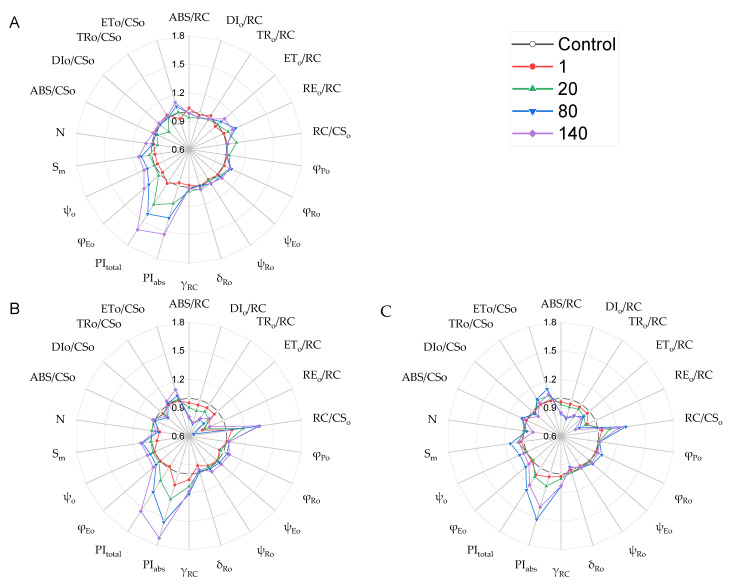
Specific energy fluxes per RC, phenomenological fluxes per CS flux ratio, and parameters of *I. pseudacorus* treated with GO for (**A**) 1 week, (**B**) 8 weeks, and (**C**) 15 weeks.

Among the selected chlorophyll *a* fluorescence parameters in this study, the performance indexes (PI_ABS_ and PI_total_) were the indicators with most noticeable changes observed in GO-treated plants. They were markedly higher than those of the control, except for in those treated with GO at 1 mg·L^−1^, whatever the duration of the treatment, with increases of 11–51% (Figure 7). The performance index can precisely reflect the state of the photosynthetic apparatus, and higher values indicate greater energy conversion efficiency of PSII [36]. Hence, GO can boost the conversion efficiency of solar energy into chemical energy of PSII. For the quantum yields of photoinduced electron transport reaction at different parts of the electron transport chain, φ_Po_ did not obviously change in GO-treated plants, while φ_Eo_, φ_Ro_, and S_m_ in plants with exposure to higher GO concentrations were evidently increased. Regarding the electron transfer efficiency, there was no notable difference in ψ_Ro_ and δ_Ro_ between GO treatments and the control, whereas GO at higher concentrations induced higher ψ_Eo_ and ψ_o_. The characteristics of the changes in quantum yields and electron transfer efficiency indicated that GO had a promoting effect on electron transfer from Q_A_^−^ to beyond plastoquinone (PQ), but none on electron transfer from PQ to the PSI acceptor side [37]. Evidently, GO favored the reoxidation of Q_A_^−^ and accelerated the electron flow beyond Q_A_^−^ on the acceptor side of PSII, and the size of the PQ pool was enlarged [38]. At the same time, the accumulation of Q_A_^−^ was reduced, and, thus, ROS formation induced by Q_A_^−^ accumulation may have decreased. For the per RC activity, the absorbed light energy (ABS/RC), the captured light energy (TR_o_/RC), and the energy used for electron transfer (ET_o_/RC) on the per active center were reduced in the 8th and 15th weeks, but the light energy absorption (ABS/CS_o_), capture (TR_o_/CS_o_), and transport (ET_o_/CS_o_) in the unit’s cross-sectional area increased (Figure 7B,C). This demonstrated that GO treatments are able to reduce the energy charge pressure of RC and reduce the possibility of photoinhibition [39]. The higher reaction center density (RC/CS_o_) and the relative concentration of RC chlorophyll (γ_RC_), with amplitude decreasing to a greater degree in thermally dissipated light energy (DI_o_/RC and DI_o_/CS_o_) in GO treated plants, also confirmed that GO treatments may improve light energy conversion efficiency. It can be seen that the plant, after exposure to GO, showed improved photosynthesis performance by optimizing electron transport at the acceptor side of PSII and improving the energy conversion efficiency of PSII.

### 2.6. Mineral Element Contents in I. pseudacorus

N, P, K, and micronutrients are essential mineral nutrients to support plant growth. Regarding the mineral element content in the shoots of GO-treated plants, the contents of Zn, Mg, K, and P did not differ significantly from the control; N, Cu, and Fe contents increased significantly by 49–69%, 34–84%, and 11–38%, respectively; and Mn content also increased, but the levels were significantly different from those of the control only in plants exposed to GO at 80 mg·L^−1^, with an increase of 25% (Table 2). The increase in the contents of N, Cu, and Fe which was observed in this study indicated that GO enhanced nutrient uptake and utilization in the plant, and consequently increased plant biomass production. The results are consistent with previous reports that GO noticeably improved the nutrient content in leaves of *Aloe vera* [40], and promoted upward translocation of Fe in rice [14]. The amount of N in plant leaves was strongly correlated with the photosynthetic capacity, and the photosynthetic rate was promoted by the increase in nitrogen content [41]. Fe and Cu are required cofactors of many proteins involved in photosynthetic electron transfer, and could, thus, promote chlorophyll synthesis and photosynthetic performance of plants through their synergistic interaction with other micronutrients [42,43]. This increase in N, Fe, and Cu content promoted the photosynthetic capacity of the plant, as supported by the favorable effects of GO on photosynthesis, allowing the plant to achieve greater growth. These results taken together, it can be assumed that the promotional mechanism of GO and its influence on plant growth may be attributed to the increase in uptake of N, Fe, and Cu by plants and the consequent enhancement of photosynthetic performance. Weng et al. [44] found that GO at 10–200 mg·L^−1^ did not induce significant changes in the TN contents of wheat shoots, while above 400 mg·L^−1^, it inhibited N uptake and accumulation in plants due to unhealthy root growth. A decrease in the N content white clover (*Trifolium repens*) grown in potted soil with GO at a high dose was also observed by Zhao et al. [45]. A recent study also reported that GO caused a decrease in the macronutrient and micronutrient content in alfalfa (*Medicago sativa* L.) by adhering to the surfaces of roots and hindering the entry of nutrients [46]. All of these results indicate that the effect of GO on nutrient uptake in plants is complicated and dependent on the plant type and GO concentration. The mechanism of the increase in N, Cu, and Fe uptake by plants after exposure to GO is clearly an important area for future research.

## 3. Materials and Methods

### 3.1. Materials and Treatments

Graphene oxide (GO) with 99% purity (1.4% Gel) was purchased from Shanghai ALADDIN Biochemical Technology Co. Ltd., Shanghai, China. According to previous studies, the designed concentrations of GO were 1, 20, 80, and 140 mg·L^−1^, which fell within the reported ranges of research concentrations [9,12,14,15]. The solution for different concentrations of GO was prepared by diluting the stock solution with 10% Hoagland nutrient solution. The 10% Hoagland nutrient solution without GO was set as the control.

Surface-sterilized seeds of *I. pseudacorus,* of the approximate size, were germinated to form seedlings. To ensure the consistency of the seedlings, those with uniform and good growth (fresh weight: 9.1 ± 1.2 g/plant) were selected and transferred to flasks containing 500 mL GO suspension at different concentrations for the solution culture experiment (1 plant per flask). Each treatment had four repetitions (flasks), with one seedling in each repetition. The hydroponic experiment was performed for a total of 15 weeks. The experiments were carried out under phytotron conditions (14 h light period, 25/20 °C day/night temperature, 70% relative humidity). The 10% Hoagland nutrient solution was supplemented every 24 h to the initial volume (500 mL) for each flask in order to compensate for nutrition consumption and water loss.

### 3.2. Analysis of Biomass and Photosynthetic Pigment Contents of I. pseudacorus

In the 15th week of the culture, plants were carefully removed from the flasks and washed using deionized water, and the whole plants were weighed (fresh weight) using a digital balance scale (with precision of ±0.01 g). Then, plant samples were dried to a constant mass in an oven at 65 °C for 48 h for dry weight measurements. The relative growth rate (RGR), based on the plants’ fresh weights, was calculated according to the following equation:

RGR (mg·g^−1^·d^−1^) = (lnW_1_-lnW_0_)/Δt [W_0_: initial fresh weight; W_1_: fresh weight at week 15; Δt = 105 day (15 weeks)] (1)

The chlorophyll and carotenoid contents of the fresh leaf disks were determined according to a previously described method [45]. Fresh leaves (0.05 g) were homogenized in 8 mL 95% ethanol for 24 h to extract chlorophylls and carotenoids. The extract was centrifuged at 1000 g for 10 min, and absorbance was determined at 665, 649, and 470 nm using an enzyme-labeled meter. The photosynthetic pigment contents were expressed as mg g^−1^ fresh weight.

### 3.3. Measurement of Chlorophyll a Fluorescence in I. pseudacorus

Chlorophyll *a* fluorescence in each plant was measured using a Handy-PEA (Hansatech Instruments Ltd., King’s Lynn, UK) at 7-week intervals during the entire experimental period, specifically, in the 1st, 8th, and 15th weeks, respectively. For each treatment, 12 measurements (4 replicates, 3 leaves in each replicate) were made after 30 min of dark adaptation. The measurement method and the selected JIP-test parameters were described in our previous work [47].

### 3.4. Analysis of Nutrient Levels of I. pseudacorus

The nutrient levels in the plant leaves were analyzed with the intent of understanding the effects of GO on nutrient uptake and utilization by *I. pseudacorus*. The N, P, K, Cu, Zn, Fe, Mn, and Mg contents in the dried leaves were determined according the methods of Zhao et al. [45]. The dry leaf samples (0.5 g) were digested with concentrated H_2_SO_4_ (98%) and H_2_O_2_ (30%), and the TN and TP concentrations of the plant were determined by the Kjeldahl method and by the molybdenum–antimony anti-spectrophotometry method, respectively. The K, Cu, Zn, Fe, Mn, and Mg levels (0.5 g dry samples) were measured by inductively coupled plasma mass spectrometry (ICP-MS 7900, Agilent Technologies, Waldbronn, Germany) after digestion by a mixture of concentrated HNO_3_ (67%) and H_2_O_2_ (30%) on an electric hot plate at 120–150 °C.

### 3.5. Data Analysis

The experimental data were presented as the means ± SDs (standard deviations). Charts were generated using the OriginPro 2021 software. The significance of the difference between means was determined by Duncan’s test at the 0.05 probability level using the SAS 9.4 software.

## 4. Conclusions

GO at 20–140 mg L^−1^ was obviously able to promote the growth of *I. pseudacorus* in terms of biomass production. In the presence of GO, the plant displayed a better ability to adjust the photosynthetic electron transfer at PSII acceptor side. The increased electron transfer ability beyond Q_A_ and limited electron transfer from P_680_ to Q_A_ directly reduced the accumulation of Q_A_^−^. Moreover, the activity of OEC, energetic connectivity or grouping of PSII units, light energy conversion efficiency, and photosynthesis performance were enhanced. In addition, GO increased plant nutrients, as indicated by the contents of N, Cu, and Fe in the plant shoots. These results suggest that GO, at selected levels, exhibits positive effects on growth of the emergent plant by promoting photosynthesis and improving plant nutrients, and shows significant potential for applications in phytoremediation of water pollution by *I. pseudacorus*. The mechanism of its promoting effect on photosynthesis performance and nutrient uptake of the plant, as well as the correlation of the two, require an in-depth exploration in future research.

## Figures and Tables

**Table 1:** Effects of GO on contents of photosynthetic pigments in leaves of *I. pseudacorus*.

GO Concentration mg·L^−1^	Chlorophyll *a* mg·g^−1^	Chlorophyll *b* mg·g^−1^	Chlorophyll *a* + *b* mg·g^−1^	Chlorophyll *a*/*b*	Carotenoid mg·g^−1^
0	0.3805 ± 0.0223 ^c^*	0.2282 ± 0.0079 ^c^	0.6087 ± 0.0299 ^c^	1.6665 ± 0.0452 ^c^	0.0502 ± 0.0038 ^c^
1	0.4508 ± 0.1008 ^bc^	0.2434 ± 0.0303 ^c^	0.6942 ± 0.1312 ^bc^	1.8345 ± 0.1865 ^bc^	0.0658 ± 0.0217 ^bc^
20	0.5790 ± 0.0905 ^b^	0.2871 ± 0.0248 ^b^	0.8662 ± 0.1153 ^b^	2.0072 ± 0.1517 ^b^	0.0931 ± 0.0207 ^b^
80	0.7966 ± 0.1488 ^a^	0.3446 ± 0.0480 ^a^	1.1412 ± 0.1967 ^a^	2.3001 ± 0.1097 ^a^	0.1396 ± 0.0331 ^a^
140	0.7305 ± 0.0381 ^a^	0.3263 ± 0.0142 ^ab^	1.0569 ± 0.0519 ^a^	2.2379 ± 0.0345 ^a^	0.1304 ± 0.0151 ^a^

* Different letters indicate significant differences between different GO concentrations according to the Duncan’s test (*p* < 0.05). All data are expressed as means ± SD (n = 4).

**Table 2:** Effects of GO on mineral element contents in shoots of *I. pseudacorus*.

GO Level mg L^−1^	Cu µg g^−1^	Fe mg g^−1^	Zn µg g^−1^	Mg mg g^−1^	Mn mg g^−1^	K mg g^−1^	N mg g^−1^	P mg g^−1^
0	1.32 ± 0.23 ^c^*	8.44 ± 0.62 ^b^	9.75 ± 1.82 ^a^	3.60 ± 0.30 ^ab^	0.29 ± 0.02 ^b^	18.95 ± 0.50 ^a^	6.01 ± 0.86 ^b^	1.80 ± 0.19 ^a^
1	1.78 ± 0.37 ^bc^	9.34 ± 1.59 ^ab^	11.91 ± 0.45 ^a^	3.80 ± 0.11 ^ab^	0.33 ± 0.05 ^ab^	19.99 ± 2.13 ^a^	8.95 ± 1.21 ^a^	1.99 ± 0.38 ^a^
20	1.93 ± 0.27 ^b^	11.02 ± 0.98 ^a^	10.99 ± 1.39 ^a^	3.99 ± 0.34 ^a^	0.32 ± 0.04 ^ab^	19.78 ± 3.44 ^a^	9.76 ± 0.99 ^a^	2.06 ± 0.17 ^a^
80	2.42 ± 0.23 ^a^	11.67 ± 2.06 ^a^	10.81 ± 3.04 ^a^	3.96 ± 0.26 ^a^	0.37 ± 0.04 ^a^	18.23 ± 2.49 ^a^	10.12 ± 1.41 ^a^	2.00 ± 0.34 ^a^
140	2.44 ± 0.28 ^a^	10.09 ± 1.24 ^ab^	11.56 ± 1.25 ^a^	3.41 ± 0.30 ^b^	0.34 ± 0.01 ^ab^	17.48 ± 3.09 ^a^	9.41 ± 0.08 ^a^	1.90 ± 0.21 ^a^

* Different letters indicate significant differences between different GO concentrations according to Duncan’s test (*p* < 0.05). All data are expressed as means ± SD (n = 4).

## Data Availability

Not applicable.
